# FACE Analysis as a Fast and Reliable Methodology to Monitor the Sulfation and Total Amount of Chondroitin Sulfate in Biological Samples of Clinical Importance

**DOI:** 10.3390/molecules19067959

**Published:** 2014-06-12

**Authors:** Evgenia Karousou, Athanasia Asimakopoulou, Luca Monti, Vassiliki Zafeiropoulou, Nikos Afratis, Panagiotis Gartaganis, Antonio Rossi, Alberto Passi, Nikos K. Karamanos

**Affiliations:** 1 Department of Surgical and Morphological Sciences, University of Insubria, 21100 Varese, Italy; 2 Laboratory of Biochemistry, Department of Chemistry, University of Patras, 26504 Patras, Greece; E-Mails: athanasia_asimakopoulou@yahoo.gr (A.A.); vickzaf@yahoo.gr (V.Z.); nafratis@upatras.gr (N.A.); pgartaganis@yahoo.gr (P.G.); 3 Department of Molecular Medicine, Unit of Biochemistry, University of Pavia, 27100 Pavia, Italy; E-Mails: luca.monti02@universitadipavia.it (L.M.); antrossi@unipv.it (A.R.)

**Keywords:** glycosaminoglycans, chondroitin sulfate, hyaluronan, fluorophore-assisted carbohydrate electrophoresis, capillary electrophoresis, high performance liquid chromatography

## Abstract

Glycosaminoglycans (GAGs) due to their hydrophilic character and high anionic charge densities play important roles in various (patho)physiological processes. The identification and quantification of GAGs in biological samples and tissues could be useful prognostic and diagnostic tools in pathological conditions. Despite the noteworthy progress in the development of sensitive and accurate methodologies for the determination of GAGs, there is a significant lack in methodologies regarding sample preparation and reliable fast analysis methods enabling the simultaneous analysis of several biological samples. In this report, developed protocols for the isolation of GAGs in biological samples were applied to analyze various sulfated chondroitin sulfate- and hyaluronan-derived disaccharides using fluorophore-assisted carbohydrate electrophoresis (FACE). Applications to biologic samples of clinical importance include blood serum, lens capsule tissue and urine. The sample preparation protocol followed by FACE analysis allows quantification with an optimal linearity over the concentration range 1.0–220.0 µg/mL, affording a limit of quantitation of 50 ng of disaccharides. Validation of FACE results was performed by capillary electrophoresis and high performance liquid chromatography techniques.

## 1. Introduction

Chondroitin sulfate (CS) is a class of glycosaminoglycan (GAG) consisting of repeating disaccharides composed of d-glucuronic acid (GlcA), and *N*-acetyl-d-galactosamine (GalNAc). The iduronation, *i.e.*, the epimerization at C-5 of GlcA, is associated with dermatan sulfate (DS) that is also known as CS-B, whereas GlcA is the characteristic uronic acid of CS. Both CS and DS can be classified as galactosaminoglycans [1]. The CS moieties are covalently linked to a protein core, forming the proteoglycans (CSPGs) that are important macromolecules of the extracellular matrix (ECM) and the membrane cell surface. Versican and aggrecan are two CSPGs with a pivotal role in homeostasis of the tissues and the cellular functions, such as cell growth and adhesion [2]. Alterations of CSPGs synthesis is often observed in pathological conditions, such as cancer and Alzheimer’s [3,4,5].

The formation of CS chains occurs in the endoplasmic reticulum and Golgi compartments by specific glycosyltransferases [6,7]. The repetitive disaccharide region is synthesized by the alternate additions of GlcA and GalNAc through the actions of specific GlcA and GalNAc transferases [6]. The sulfation of CS at various hydroxy groups on each sugar residue is catalyzed by sulfotransferases that use the sulfate group of the 3'-phosphoadenosine 5'-phosphosulfate (PAPS), the universal donor in sulfation reactions [6]. The initial sulfation pathways are distinguished in 4-*O*-sulfation and 6-*O*-sulfation of GalNAc, even though sulfation occurs also at C-2 and/or C-3 of GlcA and/or C-2 of IdoA, to yield enormous structural diversity. In mammals, CS is commonly sulfated at the C-4 or C-6 positions of GalNAc or non-sulfated [8,9].

The biological role of CS depends on the different combination and sequential arrangement of distinct disaccharide units. For instance, the CS composed of repeating disaccharides with both C-4 and C-6 sulfation of GalNAc has an important role in bone development [10] and viral infections [11], whereas it is increased in ovarian and pancreatic cancers, resulting in changes in neoplastic growth and cell motility [12]. Alterations of sulfation at C-4 and C-6 were also observed in atherosclerosis and vascular injuries [13], whereas that at C-4 is increased in blood serum of patients after coronary artery bypass operation [14]. This latter data indicate the clinical significance of CS sulfation in serum. for monitoring inflammatory responses and disease progression.

Many disorders, including mucopolysaccharidoses, kidney disorders, diabetes and leukemia, can be observed after analysis of CS/DS in urine of patients [15,16,17,18,19]. Among them, spondyloepiphyseal dysplasia Omani type, a recessive chondrodysplasia with a defect in the macromolecular sulfation pathway, showed alteration of GAG sulfation in the urine of a patient [20]. Diastrophic dysplasia (DTD) is a recessive chondrodysplasia caused by mutations in the SLC26A2 gene encoding for a widely distributed sulfate-chloride antiporter of the cell membrane [21]. Sulfate uptake impairment caused by the mutations results in undersulfation of articular and growth plate cartilage of the dtd mouse, an SLC26A2 knock-in model that recapitulates essential aspects of human diastrophic dysplasia [22,23].

Taking into consideration these findings in respect to the role of CS in pathologies, it is obvious that a validated method for the isolation and analysis of GAGs from biologic material could be a useful tool for diagnosis and monitoring a disease course. However, the low quantity of biological samples obtained from surgical operations and biopsies or samples from body liquids, such as blood serum and urine, and the high heterogeneity of the samples make it difficult to analyse CS. Analysis of derived unsaturated disaccharides from intact GAGs after specific enzymic degradation provides a practical approach to the characterization of their structures. Various strategies and methods may be used to determine unsaturated disaccharides, such as high-performance liquid chromatography (HPLC) [24,25,26], mass spectrometry (MS) [27,28] and capillary electrophoresis (CE) [29,30,31]. Reaction of unsaturated disaccharides with a fluorophore, such as 2-aminoacridone (AMAC), gave rise to higher sensitivities using either UV-Vis or fluorescence detectors. During the last decade, polyacrylamide gel electrophoresis of fluorophore labeled-saccharides (PAGEFS) (known with the commercial name of fluorophore-assisted carbohydrate electrophoresis (FACE) [32] has been used for the analysis of many samples contemporarily [33,34,35,36].

In this report, a modified sample treatment protocol and a FACE analysis set-up were applied to hyaluronan- and CS-containing human and animal fluids and tissues. Specifically, the human biologic samples used in the present study were from blood serum before and after coronary artery bypass surgery and lens capsules from healthy and with exfoliation syndrome (XFS) donors in the presence of the pharmaceutical preparation Viscoat^®^. The XFS is an age-related disorder that leads to eye pathologies, such as open angle glaucoma and cataract [37], and is characterized by an ECM alteration, including an increased expression of biglycan and hyaluronan [25,38]. Moreover, we used murine samples of urine obtained from wild type and with dtd mice for the analysis of CS amount. The analytical methods for the extraction of GAGs were focused on the enrichment of biologic samples with GAGs and the highest possible repeatability. The Δ-disaccharides obtained from purified GAG after enzymic digestion with specific lyases, were derivatized with AMAC and analysed by FACE. For the validation of this technique, samples were analysed also with the well known and established HPLC and CE techniques that, apart from the quantification option, provide the in-house diode array UV-Vis spectra or fluorescence identification platform. The linearity curves for the different standard CS amounts analysed by FACE, showed a high selectivity of this method for CS obtained from biologic samples as well as an accurate and fast analysis. Therefore, we suggest that the presenting protocol of CS extraction combined with the FACE analysis could be easily used as a fast and reliable methodology to monitor the sulfation and total amount of CS in biological samples. Moreover, the present set-up offers the significant advantage of the simultaneous analysis of at least sixteen samples contemporaneously, which is a useful tool for the comparison of biologic samples with clinical importance.

## 2. Results and Discussion

### 2.1. Sensitivity and Linearity of CS Analysis by FACE

The PAGEFS technique, widely known with the commercial name of FACE, is the electrophoretic technique for the analysis of unsaturated disaccharides after derivatization with a fluorophore, such as AMAC. This technique exploits the fluorescence of AMAC for high sensitivities and the advantages of gel electrophoresis, such as the contemporaneous analysis of at least sixteen different samples in one apparatus at the same time.

**Figure 1:**
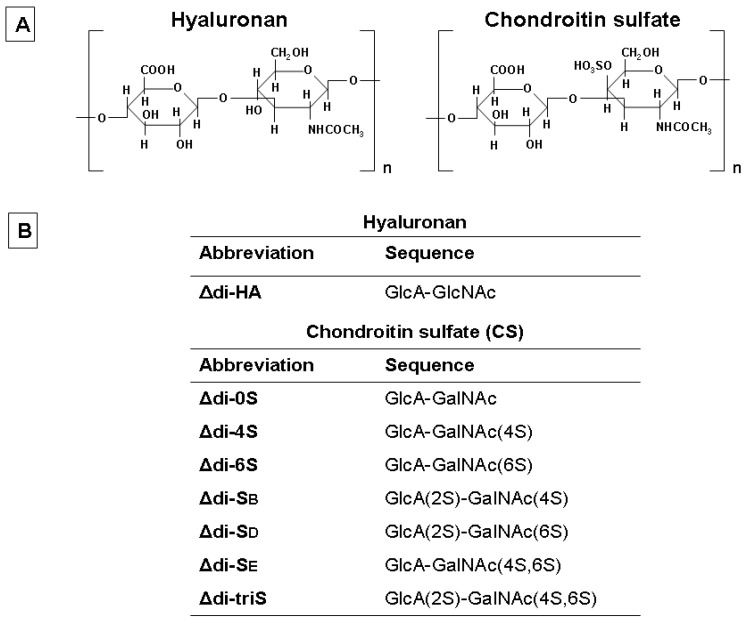
Typical structure of disaccharide units in hyaluronan and CS. Panel **A**: The backbone of hyaluronan (**left**) and CS (**right**) chains is a linear polymer composed of repeating disaccharide units, [-4GlcAβ1–3GlcNAcβ1-] and [-4(IdoAalpha/GlcAbeta)1–3GalNAcβ1-], respectively. Panel **B**: Abbreviations and sequences of typical disaccharide units of hyaluronan and CS.

Here, we present the applications of a developed FACE methodology for the analysis of hyaluronan and the various sulfated CS/DS disaccharides (Figure 1, Panel A) derivatized with AMAC, in biologic samples of clinical importance. This method is based on a combination of two different buffers for the preparation of gels and the running buffer, as well as a very precise concentration of acrylamide and *N-N'*-methylenebisacrylamide in the stacking and resolving gel, as reported previously [34,35,39]. This latter parameter presupposes the preparation of stock solutions of acrylamide by adding the solid acrylamide and *N-N'*-methylenebisacrylamide in water for final concentrations of T 50%/C 15% and T 50%/C 7.5%, where T% indicates the % (w/v) of total monomers of acrylamide and *N-N'*-methylenebisacrylamide in 100 mL of water, whereas C% indicates the % (w/w) of *N-N'*-methylenebisacrylamide in 100 g of total acrylamide monomers. These two stock solutions are necessary for the preparation of the two different parts of gel, stacking and separation, which have different proportions of T %/C % to each other. Stacking gel has a concentration of T 5%/C 1.5% which corresponds to a 1:10 dilution of T 50%/C 15% stock solution, whereas resolving gel with acrylamide concentration of T 25%/C 3.75% is prepared with a dilution of 1:2 of the T 50%/C 7.5% stock solution. A second parameter to take into consideration is the mix of Tris-borate and Tris-HCl buffers for the preparation of gels, as shown in Experimental Section, which allows separation of sulfated disaccharides as well as the separation of the non-sulfated CS and hyaluronan disaccharides.

**Figure 2:**
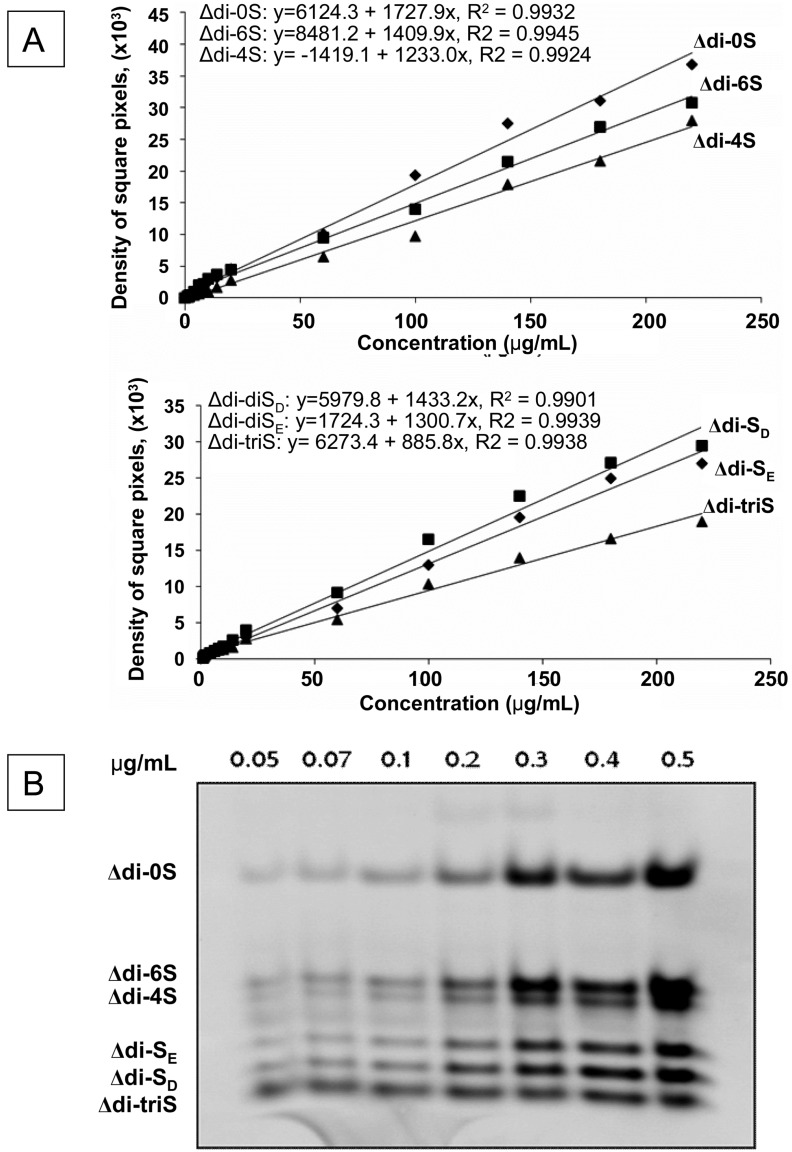
Linearity and sensitivity of FACE analysis. Panel **A**: Standard curves for Δdi-0S, Δdi-6S, Δdi-4S, Δdi-S_D_, Δdi-S_E_ and Δdi-triS CS disaccharides at the concentration levels 1.0–220.0 μg/mL obtained following derivatization with AMAC and FACE analysis. The linearity of Δ-dissacharides values was approved with *R*^2^ > 0.99. Panel **B**: Analysis of mix of standard AMAC-derived disaccharides by FACE with amounts that vary from 0.05 μg to 0.5 μg for each type of disaccharides.

A mix of non-sulfated, mono-sulfated, disulfated and tri-sulfated (Figure 1) AMAC-derived CS/DS disaccharides at various concentrations were analysed by FACE. As shown in the panel A of Figure 2, standard disaccharides of concentration level range between 1.0–220.0 μg/mL exhibited a great linearity with *R*^2^ > 0.99 with a high sensitivity of this technique that may detect as low as 50 ng of AMAC-derived disaccharides (Figure 2, Panel B), which corresponds to 25 ng or approximately 50 pmols of each type of disaccharide in 5 μL of sample loaded to the gel.

Various methods have been used for the analysis of CS. Among them, HPLC is the technique with the most developed methods for the analysis of oligo- and/or disaccharides of GAGs based on different kind of column, such a C18 of reversed phase or an anion-exchange column [40,41] or based on different detector systems, from the simple UV detector to a more sophisticated MS [33]. Recently, an established method of HPLC using turbo ion-spray ionization tandem MS showed a very high sensitivity of this method with a limit of detection for DS disaccharides at 17 ng/mL. In the last decade, methods using CE technique were described [42]. CE analysis has the advantage of the very low consumption of biologic sample and buffers and the lower time of analysis compared to HPLC. However, both HPLC and CE need at least 30 min and 10–15 min, respectively, for running one sample making the analysis of a great number of samples time-consuming [40,43]. FACE or PAGEFS using mini-slabs gels and a power pack, that is commonly found in a laboratory of biochemistry or molecular biology, is relatively low-cost, simple and fast. The great advantage of such technique is that with one apparatus, that contains two gels, the simultaneous analysis of at least sixteen samples or more, depending on the comb used for the creation of wells, may be performed in 60 min. Contrarily, CE or HPLC for the analysis of sixteen samples at least 160 or 480 min, respectively, are required [40,42]. Moreover, the analysis of various sulphated CS disaccharides is based on the comparison of the migration and the density of the bands of biologic sample to a standard mix of disaccharides run in the same gel. Contrarily to HPLC, FACE analysis requires 5 μL volume of sample [34] which means that the analysis of the same sample can be repeated using other methods. These characteristics of FACE, in addition to the high sensitivity obtained from the fluorescence of the covalently linked AMAC to the reducing end of the disaccharide, render this technique powerful for the qualitative analysis of CS chains. Moreover, using the described method of gel preparation, FACE can be used also for the simultaneous analysis of hyaluronan. In this case and for a precise quantification, it is important to treat biologic samples with specific hyaluronidases for a complete degradation of the polymer to disaccharides, as chondroitinase ABC does not completely degrade hyaluronan (data not shown).

### 2.2. Determination of Blood Serum CS in Patients before and after Coronary Bypass with FACE and CE Analysis

Extensive proteolysis and alcohol precipitation is commonly used in order to release and isolate GAGs from biologic samples [44]. In the present study, a simple sample pre-treatment procedure based on the same basic principles but with fine-tuning of all details is suggested. Blood serum samples of 150 μL were treated with protease from *Streptomyces griseus* [45]. Digestion of samples with papain was also tested but *Streptomyces griseus* protease was superior in terms of incubation temperature (37 *vs.* 60 °C) and purity of final samples that showed higher GAG amounts and reproducibility (data not shown). Samples were then boiled for a minute in the presence of high salt concentration in order to fully dissociate any interacting molecule and precipitate the bulk of proteins in serum [46]. Alcohol addition in the presence of sodium acetate leads to precipitation of the released GAGs, which are then redissolved and thus purified so as to enable the complete lysis to the constituent disaccharides by treatment with a mixture of specific chondroitinases ABC and ACII in a final volume of 50 μL [32,39]. The use of both enzymes is essential in order to fully degrade CS and DS in the corresponding disaccharides. However, by this treatment due to double bond formed between C-4 and C-5, there is no distinction between the hexuronic acid epimers of the obtained disaccharides in FACE analysis. The samples that were analyzed by FACE were first lyophilized in Speed vac and then derivatized with AMAC.

**Figure 3:**
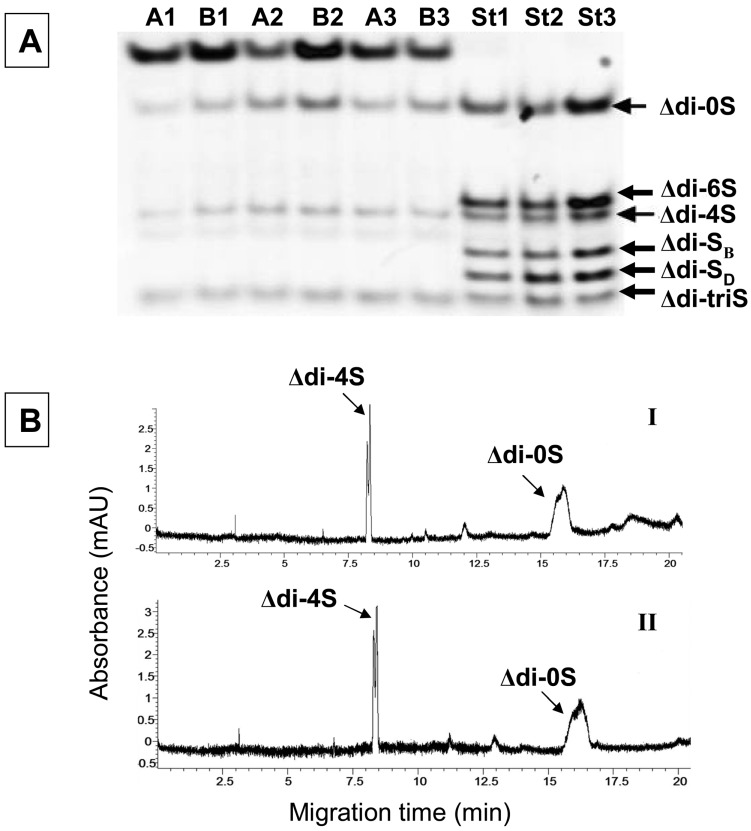
Panel (**A**): FACE analysis in blood serum samples from patients before and after coronary artery bypass surgery; the numbers 1–3 correspond to different patients and A, B in serum samples before and after surgery, respectively. The std1, std2, std3 are mixtures with standard disaccharides CS (Δdi-4S, Δdi-6S and Δdi-0S) at a concentration of 6.0 μg/mL, 8.0 μg/mL and 10.0 μg/mL, respectively. Panel (**B**): electropherograms from blood serum samples of healthy donors (I) and patients with coronary artery disease after analysis with CE analysis (II). Sulfation pattern of CS in the serum was the same in healthy and patient donors. CE analysis was performed with reverse polarity by use of 50 mM phosphate operating buffer, pH 3.0 [14].

According to FACE (Figure 3, panel A) and CE analysis (Figure 3, panel B) in blood serum of patients with coronary heart disease (CHD), it is apparent that the serum contains mainly Δdi-4S and Δdi-0S CS-derived disaccharides and traces of Δdi-6S. After quantification of each disaccharide according to standard curves for FACE and CE analysis, a significant increase of Δdi-0S disaccharides and total CS was found in blood serum samples after coronary artery bypass surgery. In contrast, there are no significant changes for the Δdi-4S disaccharides levels. Moreover, the percentage of Δdi-0S is higher in patients before bypass surgery in respect to healthy donors, which means that atherosclerotic lesions may increase the proportion of non-sulfated to sulfated CS. For each average concentration the standard deviation (SD) and coefficient of variation (CV%) are given. Moreover, FACE analysis results have no statistically significant changes from the highly sensitive and accurate CE analysis, as shown in Table 1, suggesting that FACE is also as an appropriate tool for analyzing blood serum of patients with heart diseases.

Alterations of GAG amount in blood serum could be an indication of pathology. For instance, serum hyaluronan levels were found significantly elevated diabetic mellitus individuals [47] and in women with severe malignant breast cancer associated with high metastasis in respect to the non-metastatic carcinoma or to those with benign breast disease [48]. Increase of serum CS/DS was observed in Graves’ disease [49] and in diabetes mellitus [50]. Moreover, blood and urine from aged individuals with Hurler’s syndrome was characterized by low-sulfated CS [51]. For these reasons, several studies on the analysis of GAGs in blood serum and plasma in various pathologies were presented in literature. An analytical method using LC/MS with a capillary column and ESI-MS system showed that human blood from healthy individuals contains 17 μg/mL of CS in serum and 10.4 μg/mL of CS in plasma [45]. Recently, analysis of CS by cellulose acetate electrophoresis showed that the total CS amount in healthy individuals is 10.03 mg/mL [45]. Another study on the CS disaccharides amount in human serum using HPLC technique showed a value of 9.0 ± 3.4 nmol/mL for Δdi-4S and 10.0 ± 2.9 nmol/mL for Δdi-0S, which means a total of 19 nmol/mL that corresponds to an approximately 8.7 μg/mL of total CS [40]. In our study, we found that the total CS amount analyzed by FACE is 10.44 ± 0.17 μg/mL and analyzed by CE is 10.86 ± 0.14 μg/mL. Thus, the exact amount of CS in the human blood serum of healthy individuals is still under debate. A possible reason of the different results may originate by the different dietary uses of populations and metabolic control of individuals, since it has been demonstrated that high glycemia is correlated to GAG alterations in blood [50].

**Table 1:** Comparative data of CS composition and average concentrations in the blood serum of healthy donors (*n* = 10) and patients with CHD (*n* = 6) before and after coronary artery bypass surgery after analysis with FACE and CE analytical methods.

Healthy Donors						
	**FACE**			**CE**		
**Δ-disaccharide**	**Δdi-0S**	**Δdi-4S**	**Total CS**	**Δdi-0S**	**Δdi-4S**	**Total CS**
**Mean concentration (μg/mL) ± SD**	4.22 ± 0.10	6.22 ± 0.13	10.44 ± 0.17	4.42 ± 0.10	6.44 ± 0.07	10.86 ± 0.14
**CV (%)**	2.37	2.09	1.63	2.26	1.09	1.29
**Percentage** ** (%)**	40.4	59.6		40.7	59.3	
**Before artery coronary bypass surgery**						
**Mean concentration (μg/mL) ± SD**	4.24 ± 0.12	5.38 ± 0.29	9.62 ± 0.38	4.33 ± 0.12	5.53 ± 0.18	9.86 ± 0.27
**CV (%)**	2.83	5.39	3.95	2.77	3.25	2.74
**Percentage (%)**	44.07	55.93		43.91	56.09	
**After artery coronary bypass surgery**						
**Mean concentration (μg/mL) ± SD**	5.38 ± 0.21	5.62 ± 0.21	10.99 ± 0.30	5.55 ± 0.20	5.85 ± 0.37	11.39 ± 0.46
**CV (%)**	3.90	3.74	2.73	3.60	6.32	4.04
**Percentage (%)**	48.95	51.05		48.73	51.27	

### 2.3. Determination of CS in Lens Capsules in Patients with XFS

**Figure 4:**
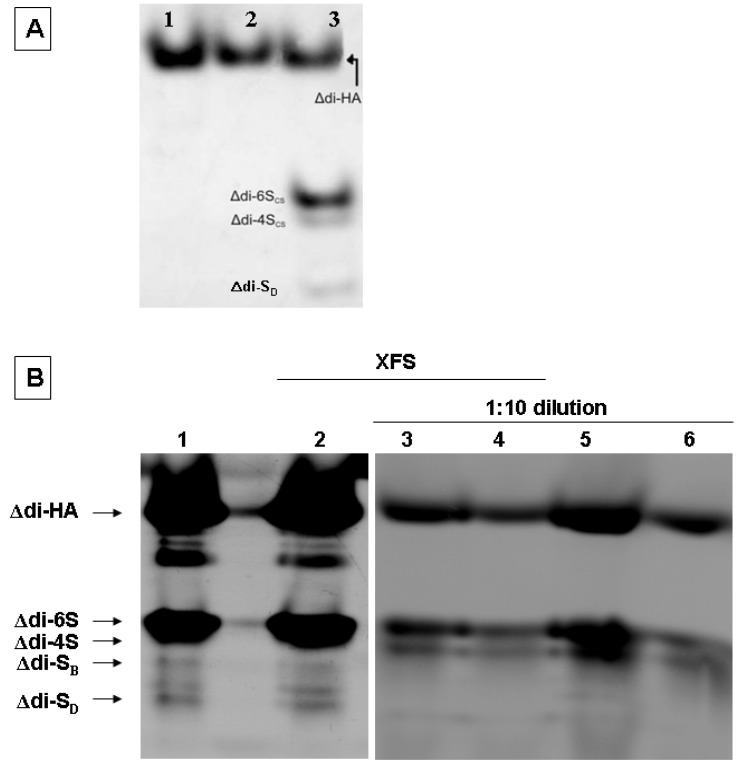
Panel (**A**): FACE analysis of high molecular weight hyaluronan (1), low molecular weight hyaluronan (2) and formulation Viscoat^®^ after enzymic digestion with chondroitinases ABC/ACII (3). Panel (**B**): FACE analysis of AMAC-derived disaccharide samples from lens capsules of patient donor with XFS before (lane 2) and after 1:10 sample dilution (lanes 3 and 4), and without XFS before (lane 1) and after 1:10 dilution (lanes 5 and 6).

The removal of the lens capsule during cataract surgery is performed with the use of a formulation that contains CS and HA (Viscoat^®^), in order to avoid eye injury. To investigate the exact CS disaccharide composition of Viscoat^®^, the formulation was first treated with chondroitinase ABC/ACII and then a part was derivatized with AMAC and analyzed by FACE analysis, whereas a second part of the obtained disaccharides was analyzed by CE without any derivatization. Results obtained from FACE analysis showed that the pharmaceutical formulation contains high amounts of Δdi-HA and Δdi-6S disaccharides and low amounts of Δdi-S_D_ and Δdi-4S (Figure 4, panel A). Although Viscoat^®^ sample was not treated with hyaluronidase, the presence of Δdi-HA was due to the treatment with chondroitinase ABC which also recognizes hyaluronan. Identification of Δdi-HA disaccharides was performed by running in the same gel standard mix of AMAC-derived HA disaccharides obtained after enzymic digestion with hyaluronidase from high and low molecular weight hyaluronan (Figure 4, panel A, lanes 1 and 2, respectively). The stronger band of Δdi-HA in lane 1 respect to lane 2 demonstrates the higher amount of disaccharides obtained from the large size polymer of hyaluronan indicating that the present FACE method can be used for the contemporaneous analysis of hyaluronan and CS. Nevertheless, Viscoat^®^ and lens capsules samples were digested only with CS lyases, as this work was concentrated on the analysis of the sulfation pattern of CS in biological samples by FACE.

Samples from lens capsule were obtained during cataract surgery in the presence of the formulation from patients with or without XFS. For convenience, samples from patients without XFS are considered as controls. FACE analysis from four samples of lens capsule are shown in Figure 4, panel B. Because of the high amounts of hyaluronan and Δdi-6S in Viscoat^®^, the loading of 5 μL of sample obtained from of 50 μL, which is the final volume after derivatization, gave strong bands for Δdi-HA and Δdi-6S for both patients with XFS (Figure 4, panel B, lane 1) or without XFS (Figure 4, panel B, lane 2), covering other bands and making the quantification of band density difficult. For this reason, the samples were diluted in a 1:10 final concentration and 5 μL volume of each sample was loaded in the gel. In this case, Δdi-6S CS and Δdi-4S were separated well showing almost the same pattern between patients with XFS (lanes 3 and 4) and without XFS (lanes 5 and 6). However, dilution of samples could not provide the identification of the disulfated disaccharides, suggesting that the analysis of both diluted and non-diluted samples should be performed.

**Figure 5:**
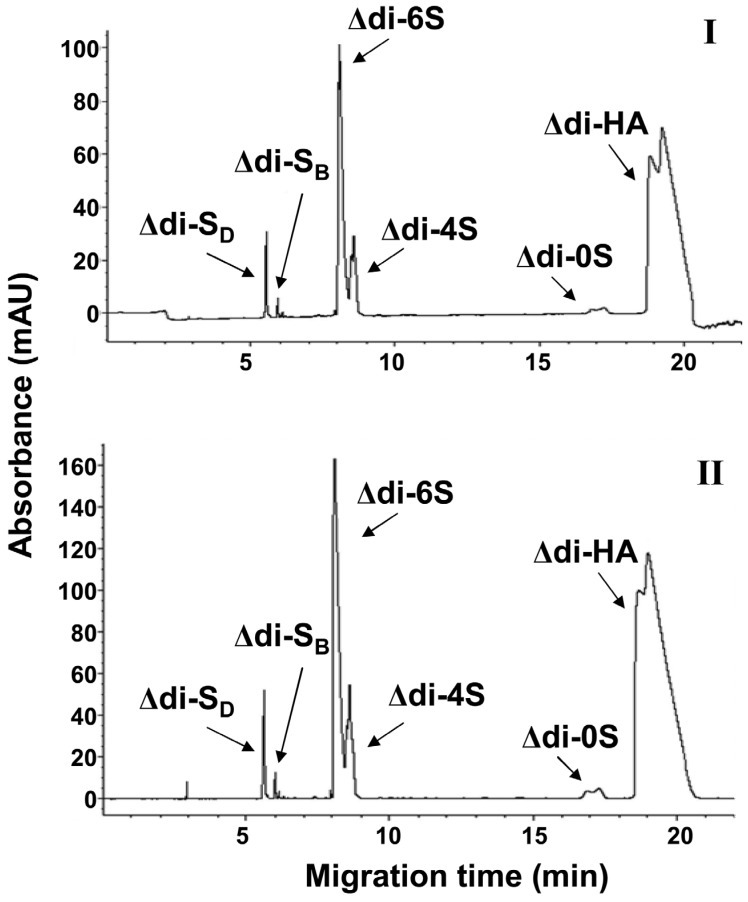
CE analysis of lens capsule from patients with cataract without XFS (**I**) and with XFS (**II**), both contained Viscoat^®^. The analysis was performed by reverse polarity and the detection of Δ-disaccharide was at 230 nm. The operating buffer was 50 mM phosphate, pH 3.0. Lens capsule of a patient with XFS contains Δdi-S_D_, Δdi-S_B_, Δdi-6S, Δdi-4S, Δdi-0S and Δdi-HA disaccharides.

In order to validate results from FACE analysis the samples from lens capsule of patients with or without XFS were analyzed by CE analysis, according to the previous protocols [31,34]. Results showed that the composition of lens capsule from individuals without XFS (Figure 5I) and with XFS (Figure 5II) contained Δdi-HA, Δdi-diS_D_, Δdi-diS_B_, Δdi-6S and Δdi-4S in a similar pattern to FACE analysis results, even though the Δdi-HA peak was not well separated. CE results showed also a very small peak, which corresponds to Δdi-0S disaccharides. However, Δdi-0S disaccharides were not identified by FACE technique neither in diluted nor in non-diluted samples. Most probably, the high level of Δdi-HA disaccharides originated from the pharmaceutical formulation covered the Δdi-0S band, whereas after dilution the band was not visible because of the very low amount.

Summarizing the data obtained from FACE and CE analysis in Table 2, it is suggested that both techniques gave a similar average composition of CS and hyaluronan. No significant differences were observed between patients with and without XFS, but this result may come from the absorbed formulation Viscoat^®^ from lens capsule. The most predominant CS disaccharide in XFS samples was Δdi-6S with 65.0% ± 1.91%, whereas the percentage of Δdi-HA in the total GAG extraction was 34.4% ± 2.47%. The concentrations of Δdi-6S and Δdi-HA of lens capsule without pharmaceutical formulation were 16.07 ± 0.23 (μg/mL) ± SD and 4.73 ± 0.31 (μg/mL) ± SD, respectively (data not shown). This profile of GAGs in lens capsule from patients with XFS shows similarity to results from iris [52] and aqueous humour analyzed by CE and ion-pair HPLC [25,53].

**Table 2:** CS sulfation pattern, hyaluronan composition and average composition of lens capsules from individuals without (control) and with XFS. The percentage of CS disaccharides refers to the total amount of CS disaccharides, whereas the percentage of hyaluronan refers to the amount of both CS and hyaluronan disaccharides.

Control						
**Δ-disaccharide**	Δdi-HA	Δdi-0S	Δdi-4S	Δdi-6S	Δdi-diSB	Δdi-diSD
**Percentage (%)**	34.7 ± 3.18	2.0 ± 0.21	19.9 ± 1.34	64.0 ± 2.69	1.7 ± 0.28	9.4 ± 0.49
**CV (%)**	9.16	10.50	6.73	4.20	16.47	5.21
**Patients with XFS**						
**Percentage (%)**	35.6 ± 2.33	1.7 ± 0.21	22.5 ± 2.12	61.3 ± 1.63	1.7 ± 0.21	9.8 ± 0.35
**CV (%)**	6.54	12.35	9.42	2.66	12.35	3.57

### 2.4. Determination of CS in Murine Urine

Extraction of CS and analysis of the derived disaccharides by FACE were applied also for urine samples obtained from a dtd murine model. The dtd mouse is a “knock-in” for a c1184t transition causing an A386V substitution in the eighth transmembrane domain of the SLC26A2, which strongly reduces the activity of the transporter and causes undersulfation of glycosaminoglycans in cartilage. Homozygous mutant mice show a chondrodysplastic phenotype that recapitulates essential aspects of human DTD [23].

**Figure 6:**
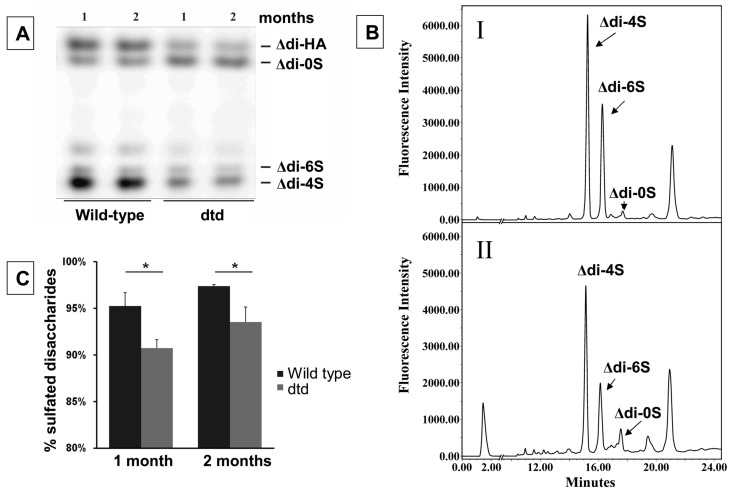
Panel **A**: FACE analysis of CS disaccharides derivatized with AMAC from urine samples of mutant and wild-type animals at 1 and 2 months of age. Panel **B**: RP-HPLC chromatogram of Δ-disaccharides from urine of a wild type (**I**) and dtd (**II**) mouse at 1 month of age. Analysis were performed with a Prontosil C18 column 4.6 × 200 mm (Bischoff) and AMAC derivatives were detected with a fluorescence detector (λex = 425 and λem = 525 nm). Panel C: Sulfated disaccharide content (Δdi-4S and Δdi-6S) relative to Δdi-0S, Δdi-4S and Δdi-6S determined by HPLC analysis of urine from mutant and wt animals at 1 and 2 months of age. GAGs in mutants are undersulfated compared to wild-type littermates. * *p* < 0.0001.

Murine urine samples from wild-type and dtd mice were collected and frozen in −20 °C until use. Macroscopically, the volume of urine samples from mutant mice with reduced skeletal growth was lower than that of the wild-type mice. For this reason, a microextraction protocol was required in order to purify and digest CS GAGs starting from 100 µL urine samples. The recovered GAGs from urine samples were digested with chondroitinase ABC and ACII and released CS disaccharides were derivatized with AMAC and analyzed by FACE and by HPLC, as described in the Experimental Section. The sulfation pattern and concentration of the analyzed disaccharides obtained from FACE (Figure 6, panel A) showed the presence of hyaluronan and non-, 4- and 6-sulfated CS disaccharides, with an evidently strong band of Δdi-4S disaccharide in wild-type mice. Contrarily, in dtd mice the sulfated CS disaccharide bands, *i.e.*, Δdi-4S and Δdi-6S, had a lower density respect to wild-type, whereas the non-sulfated Δdi-0S band density was slightly increased. The same pattern of sulfation was also presented by HPLC analysis (Figure 6, panel B). Overall, at both age points urine CS GAGs in mutant animals were undersulfated compared to wild-type littermates (Figure 6, panel C). Similar findings of GAGs undersulfation were also detected in the cartilage of dtd mice [23], demonstrating that DTD pathology leads to an overall decrease of CS sulfation and that CS analysis might be used as a biological marker of this pathology; furthermore, it could be a non invasive useful marker to follow the efficacy of any therapeutic treatment. Therefore, the established method for the extraction of CS and the FACE analysis of AMAC-derived unsaturated CS disaccharides are indicated for the analysis of CS obtained from very low volumes of urine.

## 3. Experimental

### 3.1. Chemicals and Biological Materials

Avian CS (average molecular size 18 kDa) was kindly offered by Pierre Fabre Laboratories (Castres, France). Blood sera from healthy donors and from patients with coronary artery disease before and after coronary bypass surgery were obtained from Prof. N. Tsilimingas and Dr. D. Karangelis from the Department of Cardiovascular and Thoracic Surgery, University of Thessaly, Larissa, Hospital of Thessaly, Larissa, Greece. Samples of lens capsule from healthy and with exfoliation syndrome (XFS) donors during cataract surgery were provided by Prof. S. Gartaganis in the Eye Clinic of the University of Patras, Greece. Murine urine samples used here were from male wild-type and dtd mice (*n* = 7) 1 and 2 month-old with a mixed C57Bl/6J × 129/SV background. Animals were bred with free access to water and standard pelletted food. Care and use of mice for this study were in compliance with relevant animal welfare guidelines approved by the Animal Care and Use Committee of the University of Pavia.

Unsaturated Chondro-Disaccharide Kit (C-Kit) and Unsaturated Dermato/Hyaluro-disaccharide Kit (D-Kit) (for HPLC), containing 2-acetamido-2-deoxy-3-*O*-(β-d-gluco-4-enepyranosyluronic acid)-d-glucose (Δdi-HA), 2-acetamido-2-deoxy-3-*O*-(β-d-gluco-4-enepyranosyluronic acid)-d-galactose (Δdi-0S), 2-acetamido-2-deoxy-3-*O*-(β-d-gluco-4-enepyranosyluronic acid)-4-*O*-sulfo-d-galactose (Δdi-4S), 2-acetamido-2-deoxy-3-*O*-(β-d-gluco-4-enepyranosyluronic acid)-6-*O*-sulfo-d-galactose (Δdi-6S), 2-acetamido-2-deoxy-3-*O*-(2-*O*-sulfo-β-d-gluco-4-enepyranosyluronic acid)-d-galactose (Δdi-UA2S), 2-acetamido-2-deoxy-3-*O*-(2-*O*-sulfo-β-d-gluco-4-enepyranosyluronic acid)-4-*O*-sulfo-d-galactose (ΔDi-S_B_), 2-acetamido-2-deoxy-3-*O*-(2-*O*-sulfo-β-d-gluco-4-enepyranosyluronic acid)-6-*O*-sulfo-d-galactose (ΔDi-S_D_), 2-acetamido-2-deoxy-3-*O*-(β-d-gluco-4-enepyranosyluronic acid)-4,6-di-*O*-sulfo-d-galactose (ΔDi-S_E_), 2-acetamido-2-deoxy-3-*O*-(2-*O*-sulfo-β-d-gluco-4-enepyranosyluronic acid)-4,6-di-*O*-sulfo-d-galactose (ΔDi-triS), were purchased from Seikagaku Corporation (Tokyo, Japan). Protease from *Streptomyces griceus* (Cat. No. 537088) was obtained from Calbiochem (Darmstadt, Germany). Chondroitinases ABC (EC 4.2.2.4) from *Proteus vulgaris* and AC II (EC 4.2.2.6) from *Arthrobacter aurescens* were from Seikagaku Corporation. 2-Aminoacridone (AMAC) and NaBH_3_CN were from Sigma-Aldrich (Steinheim, Germany). All solutions were prepared using ultrapure water MilliQ or prepared by purifying deionised water to attain a sensitivity of 18 MΩ cm at 25 °C.

### 3.2. Procedure for Isolation of Blood Serum CS before and after Coronary Bypass

A 20 μL volume of 50 mM Tris-HCl buffer (pH 8.0) and 5 μL of protease solution (0.08 U/10 μL) in 50 mM Tris-HCl buffer (pH 8.0) were added to 150 μL serum sample in an Eppendorf tube, and the mixture was incubated overnight at 37 °C. Then, 40 μL of a NaCl solution for a final concentration of 0.5 M per sample was added and the mixture was heated in a boiling water bath for 1 min and cooled on ice. After centrifugation at 12,000 rpm for 5 min, the supernatant was transferred to an eppendorf tube and four volumes of ethanol (95%) saturated in sodium acetate were added. All samples were stored at 4 °C for 2 h, to allow precipitation of GAGs, and were centrifuged at 12,000 rpm for 5 min. The precipitate was then dissolved in 50 mM Tris-HCl buffer, pH 7.5, and was treated with 5 μL of a mixture of chondroitinases ABC and ACII (0.05 unit each, final volume of 50 μL) for 3 h at 37 °C. Following centrifugation at 12,000 rpm, the supernatants were analyzed either by CE or were lyophilized, derivatized with AMAC and finally were analyzed by the FACE technique.

### 3.3. Isolation of CS and HA from Lens Capsules

Samples of lens capsules were received during cataract surgery from four different patients with XFS and four patients without XFS. Biological samples contained the pharmaceutical formulation Viscoat^®^ that contains 40 mg/mL sodium CS and 30 mg/mL sodium hyaluronate, which was injected during surgery in order to maintain a deep chamber during anterior segment surgeries and protect the corneal endothelium and other ocular tissues.

Tissues obtained from lens capsules were placed in Eppendorf tubes and a mixture of 290 μL of 50 mM Tris-HCl buffer (pH 8.0) with 0.08 U of proteases was added, following an incubation at 37 °C for 16 h. Then, 40 μL of a NaCl solution (final concentration in sample 0.5 M) was added to the lens capture solution; mixtures were heated in a boiling water bath for 1 min and cooled on ice. After centrifugation at 12,000 rpm for 5 min, the supernatant was transferred to an eppendorf tube and four volumes of ethanol (95%) saturated in sodium acetate were added. All samples were stored at 4 °C for 2 h and then centrifuged at 12,000 rpm for 5 min. The pellet was then dissolved in 50 mM Tris-HCl buffer, pH 7.5, and was treated with 5 μL of a mixture of chondroitinases ABC and ACII (0.05 unit each, final volume of 50 μL) for 3 h at 37 °C. Following centrifugation at 12,000 rpm, the supernatants were analyzed either by CE or were lyophilized, derivatized with AMAC and finally were analyzed by FACE technique.

### 3.4. CS Extraction from Mouse Urine

Genomic DNA was isolated from mouse tail clips and genotyping to distinguish homozygous mutant animals from heterozygous and wild-type littermates was then performed either by PCR or by southern blotting.

Urine samples were collected from mice and frozen at −20 °C until used. Samples were thawed and centrifuged in Eppendorf tubes at 12,000 rpm (13,000 *×g*) for 5 min to remove any insoluble material. GAGs in 100 µL of the urine supernatant were precipitated with 2 µL of 10% cetylpyridinium chloride (CPC, Sigma, final concentration 0.2% CPC) at 4 °C overnight. Samples were then centrifuged for 15 min at 12,000 rpm (13,000 *×g*) at 4 °C and the pellet was suspended in 500 µL of 10% potassium acetate (Sigma) in 96% ethanol (Merck Milan, Italy) and immediately centrifuged for 10 min at 12,000 rpm (13,000 *×g*) at RT to remove CPC; this step was repeated two additional times with 10% potassium acetate and three times with 500 μL of 96% ethanol to remove potassium acetate. The pellet was dried at RT and dissolved in 200 μL of 0.1 M ammonium acetate (Fluka Milan, Italy), pH 7.35, containing 30 mU chondroitinase ABC and 30 mU chondroitinase ACII () at 37 °C o/n. Samples were then centrifuged and CS disaccharides in the supernatant were lyophilized.

### 3.5. Derivatization

Derivatization of standard HA and CS Δ-disaccharides was performed as described previously by Calabro *et al.* (2001) [54], and modified by Mitropoulou *et al.* [31]. In brief, the samples were lyophilized to dryness and 5 μL of 0.1 M AMAC in glacial CH_3_COOH-DMSO 3:17% (v/v), 5 μL solvent CH3COOH-DMSO 3:17% (v/v) and 10 μL of fresh solution of 0.1 M NaBH_3_CN were added. The incubation of the samples was carried out at 45 °C for 4 h. Then, samples were centrifuged for 3 min at 7,000 rpm and added 30 μL DMSO 50% (v/v) to terminate the reaction. Alternatively, lyophilized samples were derivatized with AMAC as described previously [34]. Briefly, 30 µL of 12.5 mM AMAC (Life Technologies Italia, Monza, Italy) in DMSO/glacial acetic acid (85:15 v/v) were added and samples were incubated at RT for 15 min in the dark; then 30 µL of freshly prepared 1.25 M NaBH_3_CN (Sigma) in water was added followed by incubation at 37 °C in the dark overnight. Finally the samples were centrifuged for 3 min at 7,000 rpm, followed by polyacrylamide gel electrophoresis. Both procedures have high efficiency and may be used.

### 3.6. FACE Analysis

Electrophoresis was performed with the Consort EV215 system of Turnhout glass plates 7.5 cm, 0.75 cm spacer and cell is 0.5 cm, as previously described [34] and modified as follows. The stock solutions of buffers and acrylamide were the following: 1.5 M Tris-borate, pH 8.8, 1.5 M Tris-HCl, pH 8.8, acrylamide T 50%/C 15% and T 50%/C 7.5%. A volume of 10 mL of separating gel was prepared with a final concentration of acrylamide 25% T/3.75% C in a mix of buffer with concentrations 187.5 mM Tris-borate and 187.5 mM Tris-HCl, pH 8.8. The solution was degassed in a sonicator for 5 min and 5 μL TEMED were first added for creating radicals of acrylamide and then 50 μL ammonium persulfate of 10% w/v were added to enhance polymerization. Once the separating gel was polymerized, a 5 mL volume of stacking gel with final concentration of acrylamide T 5%/C 1.5% in 0.36 M Tris-HCl, pH 8.8, was prepared, following addition of 10 μL of TEMED and 50 μL of ammonium persulfate APS 10% (w/v).

Sample and standard mix were supplemented with 5 μL glycerol (20% v/v of final concentration within the sample) and 5 µL were loaded in the wells. A marker sample containing the bromophenol blue dye (0.02% w/v) was loaded in an empty well. Electrophoresis of samples was performed at 350 V at 4 °C and was completed when the dye reached about 1.2 mm from the edge of the gel (~60 min).

The gels were scanned with camera CCD and gel images were acquired by VersaDoc 3000 imaging system (BioRad Milan, Italy). Quantification of the bands was performed either by QuantityOne software (BioRad) or by Image J software, comparing the migration and the pixel intensity of a mix of 100 nmols per sample of standard AMAC-derived Δ-disaccharides running on the same gel.

### 3.7. CE Analysis

Biological samples obtained from human serum and lens capsules were also analyzed by CE, in order to evaluate the FACE analysis. CE was performed on an HP^3D^CE instrument (Agilent Technologies, Waldbronn, Germany) with a built-in diode array detector. Separation and analysis were carried out on an uncoated fused silica capillary tube (extended light path capillary, 50 μm i.d., 56 cm effective length,) at 25 °C. Before its first use, the fused silica capillary was washed with 1.0 M NaOH for 5 min, with H_2_O for 5 min, and with the operating buffer for 10 min. Before each run, the capillary tube was washed with 0.1 M NaOH for 1 min, with H_2_O for 2 min, and with the operating buffer for 5 min. After each analysis, the capillary was post-conditioned with H_2_O for 2 min. Before use, the operating buffer was filtered through a 0.2-μm membrane filter and degassed with agitation in an ultrasonic bath. The operating buffer replenishment mode of the CE instrument was used during electrophoresis and the autosampler temperature was 25 ± 4 °C.

Analysis of CS disaccharides was performed as previously reported [55] at 30 kV using reversed polarity in 50 mM phosphate buffer (pH 3.0) produced by dissolving sodium dihydrogen phosphate in water to the 50 mM concentration and adjusting the pH with a 3 M H_3_PO_4_ solution. The operating buffer replenishment mode of the CE instrument was used during electrophoresis. Samples were introduced hydrodynamically (500 mbar·s) using the pressure injection mode. Detection was performed at 232 nm. Peak areas were recorded and evaluated using the HP Kayak XA software system HP^3D^CE ChemStation.

### 3.8. HPLC Analysis

In order to evaluate FACE results of murine urine samples, the obtained AMAC-derived CS disaccharides were also analyzed by HPLC. Separation and analysis was performed using a reverse-phase column (Prontosil 120-3-C18-ace EPS 3 µm, 4.6 × 200 mm, Bischoff, Leonberg, Germany). A gradient elution was performed using a binary solvent system composed of 0.1 M ammonium acetate (Fluka), pH 7.0, (eluent A) and acetonitrile (Merck) (eluent B). The following elution program was used: 3 min 100% eluent A, linear gradient to 15% B in 3 min, linear gradient to 25% B in 24 min, wash step with 60% B for 10 min followed by equilibration in 100% eluent A for 8 min in preparation for the next analysis. Sample volumes injected in the column ranged from 3 µL to 15 µL and the flow rate was 1 mL/min at RT. Fluorescence detection of AMAC derivatives was achieved with a Waters 2475 fluorescence detector with an excitation wavelength of 425 nm and an emission wavelength of 525 nm.

### 3.9. Statistical Analysis

All values were expressed as mean value ± standard deviation (SD). Linear regression was performed using the GraphPad Software (version 3.0). The coefficient of variation that provides a measure of the analytical variability of the measurements was expressed by the equation: CV % = (SD/x) × 100. The correlation coefficient was calculated using *t*-test. A value of p < 0.05 was considered to indicate a statistically significant difference.

## 4. Conclusions

In this report, we present a fast and reliable analytical tool based on FACE to monitor the sulfation and total amount of CS in biologic samples of clinical importance. The protocol involves the extraction of CS from low volumes of biologic fluid and tissues, such as serum, urine and lens capsule, followed by FACE analysis. The analysis of CS was based on the complete degradation of proteins, purification of total GAGs and the complete degradation of CS/DS to unsaturated disaccharides, derivatization with AMAC and a consequence FACE analysis. The results obtained were validated with HPLC and CE and compared to those from literature. The application of this analytical method has the advantage to be fast, easy and of low-cost. Although other analytical methods have greater sensitivity, FACE technique can be used for the determination of disaccharides with a low mass of 0.05 ng. Moreover, we demonstrated that this methodology is precise and accurate and has a high selectivity for CS in biological samples. In case that the biologic samples are treated with specific enzymes for the complete degradation of hyaluronan, the established FACE technique can be used for the contemporaneous separation of CS/DS and hyaluronan. Thus, FACE analysis can be a powerful tool for diagnostic purposes and/or a follow-up of pathology, since identification of low amounts of various sulfated CS disaccharides obtained from low amounts of biologic samples can be performed.
